# Activation of GPR119 Stimulates Human *β*-Cell Replication and Neogenesis in Humanized Mice with Functional Human Islets

**DOI:** 10.1155/2016/1620821

**Published:** 2016-06-16

**Authors:** Ansarullah, Colette Free, Jenica Christopherson, Quanhai Chen, Jie Gao, Chengyang Liu, Ali Naji, Alex Rabinovitch, Zhiguang Guo

**Affiliations:** ^1^The Sanford Project, Children Health Research Center, Sanford Research, Sioux Falls, SD 57104, USA; ^2^Department of Hepatobiliary Surgery, People's Hospital, Peking University, Beijing 100044, China; ^3^Department of Surgery, University of Pennsylvania, Philadelphia, PA 19104, USA; ^4^Department of Pediatrics, Sanford School of Medicine, University of South Dakota, Sioux Falls, SD 57104, USA; ^5^Department of Surgery, Sanford School of Medicine, University of South Dakota, Sioux Falls, SD 57104, USA

## Abstract

Using humanized mice with functional human islets, we investigated whether activating GPR119 by PSN632408, a small molecular agonist, can stimulate human *β*-cell regeneration* in vivo*. Human islets were transplanted under the left kidney capsule of immunodeficient mice with streptozotocin- (STZ-) induced diabetes. The recipient mice were treated with PSN632408 or vehicle and BrdU daily. Human islet graft function in the mice was evaluated by nonfasting glucose levels, oral glucose tolerance, and removal of the grafts. Immunostaining for insulin, glucagon, and BrdU or Ki67 was performed in islet grafts to evaluate *α*- and *β*-cell replication. Insulin and CK19 immunostaining was performed to evaluate *β*-cell neogenesis. Four weeks after human islet transplantation, 71% of PSN632408-treated mice achieved normoglycaemia compared with 24% of vehicle-treated mice. Also, oral glucose tolerance was significantly improved in the PSN632408-treated mice. PSN632408 treatment significantly increased both human *α*- and *β*-cell areas in islet grafts and stimulated *α*- and *β*-cell replication. In addition, *β*-cell neogenesis was induced from pancreatic duct cells in the islet grafts. Our results demonstrated that activation of GPR119 increases *β*-cell mass by stimulating human *β*-cell replication and neogenesis. Therefore, GPR119 activators may qualify as therapeutic agents to increase human *β*-cell mass in patients with diabetes.

## 1. Introduction

Insufficient functional pancreatic *β*-cell mass resulting in insulin deficiency is the underlying cause of type 1 diabetes and a major contributor to type 2 diabetes [1, 2]. Therefore, restoring and/or expanding the functional pancreatic *β*-cell mass in diabetic patients and in islet transplanted patients would have therapeutic value. Over the last decade, incretin-based therapies for *β*-cell preservation and/or regeneration have been studied [3, 4].

Glucagon like peptide-1 (GLP-1) is an incretin hormone secreted by the enteroendocrine intestinal L cells. GLP-1 binds to the GLP-1 receptor which is expressed in many cells including pancreatic islet *β*-cells and pancreatic exocrine duct cells [5]. GLP-1 binds to GLP-1R and activates adeylate cyclase, increasing cAMP levels, activating protein kinase A, increasing intracellular Ca^+2^ levels, and finally leads to stimulation of exocytosis of insulin secretory granules [6]. GLP-1 increases glucokinase activity in pancreatic *β*-cells, which phosphorylates D-glucose and enhances glucose-stimulated insulin secretion [7]. It also regulates glucose homeostasis by suppressing glucagon secretion from islet *α*-cells resulting in a reduction of hepatic glucose output. In addition, GLP-1 and its analogues are capable of inducing *β*-cell replication in murine models as well as in humans [8–10]. GLP-1 has also been reported to exert antiapoptotic properties on pancreatic *β*-cells [11–14].

G protein-coupled receptor 119 (GPR119) was identified as an orphan G protein-coupled receptor expressed in pancreatic *β*-cells and intestinal L-cell [15–18]. Activation of GPR119 by endogenous ligands or small molecules leads to accumulation of intracellular cAMP and further insulin and GLP-1 release. PSN632408, a selective small molecular GPR119 agonist, has been reported to increase intracellular cAMP levels in a GPR119 dependent manner [15]. Previously, we reported that PSN632408 increases plasma active GLP-1 levels in mice and stimulates *β*-cell replication in mouse islet grafts and in the pancreas of mice with streptozotocin- (STZ-) induced diabetes [19, 20]. Whether GPR119 agonists improve human *β*-cell function and stimulate human *β*-cell regeneration has not been addressed.

*β*-cell regeneration and its related mechanisms have been extensively studied in murine/rodent models. However, there are marked differences between rodent and human islets in relation to cytoarchitecture, cellular composition, and replication rate [10, 21–25]. Humanized mouse models have been used to study human islet *β*-cell function and regeneration* in vivo* by transplanting human islets into immunodeficient mice with STZ-induced diabetes [26–28]. We previously evaluated human islet function and regeneration in humanized mouse models treated with clinically approved antidiabetic drugs and novel drug candidates. We found that human islet grafts are fully functional since immediate increases of human C-peptide were observed following glucose challenge and postprandial glucose levels were well controlled [29]. Long-term human islet graft function was assessed by monitoring blood glucose levels in recipient mice and finding normoglycaemia over 14 weeks after transplantation. After removing the kidney bearing the human islet grafts, all recipient mice returned to hyperglycaemia within one week after nephrectomy, confirming human islet graft function. Thus, the stability of the model and the long-term survival of human islet grafts allow the study of agents and mechanisms of human *β*-cell function and regeneration* in vivo*.

We previously reported that exendin-4 (a GLP-1 analogue) can stimulate human *β*-cell replication in humanized mouse model [10]. In this study, we investigated whether a small molecular agonist of GPR119 can improve human *β*-cell function and stimulate *β*-cell regeneration in a humanized mouse model.

## 2. Materials and Methods

### 2.1. Animals

NOD.scid mice (NOD.CB17-*Prkdc* ^*scid*^/J), 8–10 weeks of age, were purchased from Jackson Laboratory (Bar Harbor, Maine, USA). These mice were acclimatized for at least 7 days in their new environment with* ad libitum* access to normal chow and autoclaved water. The mice were housed in Laboratory Animal Research Facility of Sanford Research/USD under specific pathogen-free conditions. All experiments were performed in accordance with the protocol approved by the Sanford Research/USD Institutional Animal Care and Use Committee (Protocol # 77-08-16D).

### 2.2. Diabetes Induction and Glucose Measurement

Diabetes was induced in NOD.scid mice by single intraperitoneal (IP) injection of STZ at 180 mg/kg body weight. One week after STZ treatment, nonfasting blood glucose levels were measured daily at 8.30–11.00 AM in tail vein blood using a Bayer Contour*™* Glucometer (Bayer HealthCare, Tarrytown, NY, USA). Diabetes was diagnosed when blood glucose was >400 mg/dL (22.2 mmol/L) for two consecutive days. These mice were diabetic for at least two weeks before transplantation and were treated with 0.5 U Novolin R and 0.5 U NPH insulin (Novo Nordisk, Copenhagen, Denmark) daily. Normoglycaemia after transplantation was defined as the nonfasting blood glucose level in recipient mice <200 mg/dL for two consecutive days and thereafter.

### 2.3. Islet Transplantation

Human islets from four pancreatic donors were received from the Integrated Islet Distribution Program (IIDP) of the National Institute of Diabetes and Kidney and Digestive Diseases (NIDDK) and the University of Pennsylvania, Philadelphia. Since islets were isolated from cadaveric donors and no living individuals were involved, our study did not meet the definition of research with human subjects. Sanford Health Institutional Review Board had reviewed our study and documented that our study did not meet the regulatory requirements for human subject research. The age of donors was 14.0 ± 3.6 years and BMI was 25.8 ± 2.7. The purity of islets was 72.5 ± 8.5% and the viability of islets was 91.0 ± 3.3%. At least three diabetic NOD.scid mice in each treatment group were transplanted islets from the same donor. The recipient mice were anaesthetized with isoflurane. The skin of the left lateral side was shaved and cleaned with Betadine. Using a dissecting microscope, a lumbar incision (~1.5 cm) was made perpendicular to the axis of the kidney across the left side. The left kidney was carefully pushed out through a lumbar incision by using a Q-tip. Using two small forceps, a small hole was opened in the lower half of the kidney capsule. A polyethylene tube (PE-50) containing 1500 islet equivalents (IE) was inserted beneath the kidney capsule and gently pushed from the lower pole to the upper pole. The human islets were then delivered to the upper pole of the kidney by a Hamilton syringe. The hole of the kidney capsule was closed by cautery loop. The incision was closed by using continuous 5-0 Dexon absorbable suture with tapered needle. All recipient mice received buprenorphine (0.1 mg/kg, s.c.) daily for 3 days after surgery. Nonfasting blood glucose levels were measured daily during first week after transplantation and then at least 3 times per week until the end of experiment.

### 2.4. Treatment

Starting from the day of transplantation, recipient mice were treated with vehicle (DMSO) or GPR119 agonist, PSN632408, 4-[[3-(4-pyridinyl)-1, 2, 4-oxadiazol-5-yl] methoxy]-1-piperidinecarboxylic acid, and 1,1-dimethylester (Cayman Chemicals, Ann Arbor, MI, USA) 10 mg/kg daily by gavage for 4 weeks. In addition, all diabetic mice were treated with 1.0 U of insulin daily. Insulin treatment was stopped if the blood glucose level was <200 mg/dL. To label replicating cells, all mice were injected intraperitoneally with 5-bromo-2′-deoxyuridine (BrdU) at 100 mg/kg daily for 4 weeks starting from day of transplantation.

### 2.5. Oral Glucose Tolerance Test (OGTT)

At the end of the treatment period, mice that achieved normoglycaemia underwent an OGTT. The mice were fasted overnight and blood glucose levels were measured by tail vein sampling on day 29. Glucose 2 g/kg body weight was given by gavage to each mouse. Blood glucose levels were determined at 0, 15, 30, 60, and 120 minutes after glucose administration.

### 2.6. Nephrectomy

Following OGTT, the transplanted mice were anaesthetized using isoflurane. The skin of the dorsal lumbar area side was shaved and cleaned with Betadine. A cranial caudal skin incision was made and the abdominal cavity was entered. The left kidney bearing islet grafts was lifted free from the surrounding tissue and pulled out of the incision gently. The adrenal gland was gently freed from the kidney and returned to the abdominal cavity. The renal blood vessels and ureter were ligated and transected to remove the kidney. The incision was closed with 5-0 Dexon absorbable suture. All recipient mice received buprenorphine (0.1 mg/kg, s.c.) daily for 3 days after operation.

### 2.7. Immunofluorescence and Confocal Microscopy

Human islet grafts were fixed in 4% paraformaldehyde and embedded in paraffin wax. Five-micron-thick serial sections from islet grafts were mounted on charged slides (Fisher Scientific, Pittsburgh, PA, USA). For the immunoflourescent detection of insulin/BrdU, glucagon/BrdU, amylase/BrdU, insulin/Ki67, and insulin/cytokeratin 19 (CK19, a ductal cell marker), sections were deparaffinized in Citrisolv*™* (Fisher Scientific, Pittsburgh, PA) and rehydrated with grades of alcohol. An antigen unmasking step was performed for 30 minutes in a pressure cooker using Decloaker solution (Biocare Medical, Concord, CA, USA). DNA was denatured to expose the antigen by incubating the tissue sections in 1 N HCl for 45 minutes at 37°C. The sections were washed with PBS and blocked with 5% normal donkey serum (Jackson ImmunoResearch Laboratories, West Grove, PA, USA). Sections were then incubated with their respective primary antibodies overnight at 4°C. The primary antibodies used were rat anti-BrdU monoclonal antibody (1 : 200) purchased from Accurate Chemicals (Westbury, NY, USA), guinea pig anti-insulin (1 : 200), rabbit anti-glucagon (1 : 200), rabbit anti-amylase (1 : 200), rabbit anti-CK19 (1 : 200), and rabbit anti-Ki67 (1 : 200) purchased from Abcam (Cambridge, MA, USA). Next, the labeled sections were washed with PBS and secondary antibody incubation was carried out in the dark for 45 minutes at 37°C. Fluorochrome-conjugated secondary antibodies Alexa-Fluor 488, Alexa-Fluor 546, or Alexa-Flour 647 F(ab′)_2_ (Jackson ImmunoResearch Laboratories, West Grove, PA, USA) were used at 1 : 200 dilution. 4′,6-Diamidino-2-phenylindole (DAPI) was used to visualize nuclei. Negative controls were run where the primary antibodies were omitted. Tissue sections were washed in calcium and magnesium-containing PBS and mounted with antifade mounting medium Vectasheild (Vector Laboratories, Burlingame, CA, USA). Nikon TIRF, a laser scanning confocal microscope, was used with a 63X1.4NA pan Apochromat objective with optical *Z* sections taken at ~0.8 microns. Magnification, laser and detector gains, and pinhole settings were set below saturation and were identical across samples.

### 2.8. *β*-Cell Area, Replication, and Neogenesis in Human Islet Grafts

To evaluate *β*-cell area, 5 islet graft slides (comprising 3 sections each) spanning from top to bottom of the graft at least 100 *μ*m apart per donor from each group were analyzed for insulin positive area using the lasso/ROI tool using NIS elements imaging software (Nikon Instruments Inc., Melville, NY, USA). *β*-cell area was calculated as the ratio of the insulin positive area over the total graft area. To evaluate *β*-cell replication, the ratio of insulin/BrdU copositive cells over total insulin positive cells in islet grafts sections was calculated. The ratio of Ki67 positive *β*-cells over total *β*-cells was also calculated to measure *β*-cell replication. To evaluate *β*-cell neogenesis, CK19 and insulin copositive cells in islet grafts from mice treated with vehicle (*n* = 4) and treated with PSN632408 (*n* = 9) that reversed normoglycaemia at 4 weeks after transplantation were examined. Five sections from each graft (about 50 microns apart) per animal comprising 800–1000 CK19^+^ cells were counted. The cells that were insulin and CK19 copositive and embedded within ductal cells were counted, but not the cells that were in close proximity. The ratio of CK19/insulin copositive cells over total CK19 positive cells was calculated.

### 2.9. *α*-Cell Area, Replication, and Acinar Cell Replication in Human Islet Grafts

To evaluate *α*-cell area, 5 islet graft slides (comprising 3 sections each) spanning from top to bottom of the graft at least 100 *μ*m apart per donor from each group were analyzed for glucagon positive area using the lasso/ROI tool using NIS elements imaging software (Nikon Instruments Inc., Melville, NY, USA). *α*-cell area was calculated by dividing the glucagon positive area by the total graft area. To evaluate *α*-cell replication, the ratio of glucagon/BrdU copositive cells over total glucagon positive cells in islet grafts sections was calculated. To evaluate acinar cell replication, the ratio of amylase/BrdU copositive cells over total amylase positive cells in islet grafts sections was calculated.

### 2.10. Statistics

The significance of differences between vehicle and PSN632408 treatments was determined using Kaplan Meier curve, Log Rank (Mantel-Cox) test, and Student's *t*-test with Welch's correction. The results are expressed as mean ± SEM using Graph Pad Prism version 6.0 (Graph Pad Software, San Diego, CA, USA). A value of *P* < 0.05 was considered statistically significant.

## 3. Results

### 3.1. PSN632408 Treatment Improved Human Islet Function after Transplantation

**Figure 1:**
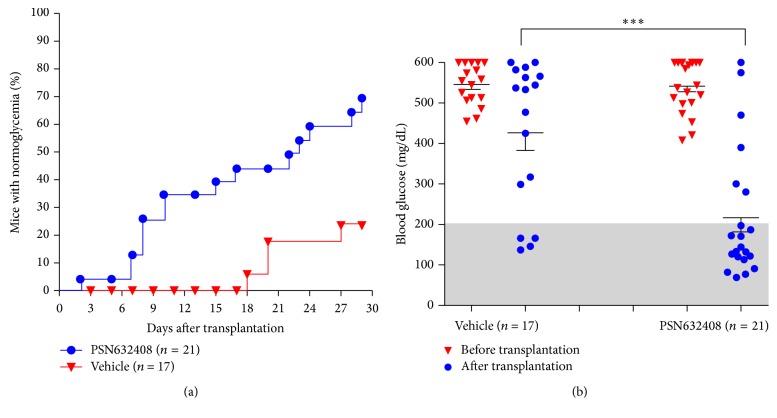
(a) Percentage of STZ-induced diabetic NOD.scid mice achieving normoglycaemia after human islet transplantation and 4 weeks of treatment with PSN632408 or vehicle. At 4 weeks after transplantation, percentage of mice with normoglycaemia was 71% in PSN632408-treated diabetic mice and 24% in vehicle-treated mice (*P* < 0.002). (b) Nonfasting blood glucose levels in PSN632408-treated mice and in vehicle-treated mice before transplantation and at 4 weeks after transplantation. PSN632408 treatment significantly reduced the mean nonfasting blood glucose level compared with vehicle treatment at 4 weeks after transplantation (^*∗∗∗*^ *P* < 0.001).

Two weeks after transplantation, 38% of PSN632408-treated recipient mice achieved normoglycaemia (*n* = 21), compared with none of vehicle-treated recipient mice (*n* = 17). Four weeks after transplantation, 71% (15 out of 21) of PSN632408-treated recipient mice achieved normoglycaemia compared with only 24% (4 out of 17) of vehicle-treated mice (*P* < 0.002) (Figure 1(a)).

Nonfasting blood glucose in PSN632408-treated mice was 542.5 ± 14.6 mg/dL before transplantation and 216.8 ± 35.1 mg/dL 4 weeks after transplantation (*P* < 0.001). In contrast, vehicle-treated recipient mice had nonfasting blood glucose levels of 545.5 ± 12.1 mg/dL before transplantation and 426.2 ± 43.5 mg/dL 4 weeks after transplantation (*P* < 0.01). Also, PSN632408-treated mice had a significant reduction in nonfasting blood glucose compared with vehicle-treated mice 4 weeks after transplantation (*P* < 0.001) (Figure 1(b)). Body weights in the mice were unchanged after transplantation and similar in PSN632408- and vehicle-treated mice.

**Figure 2:**
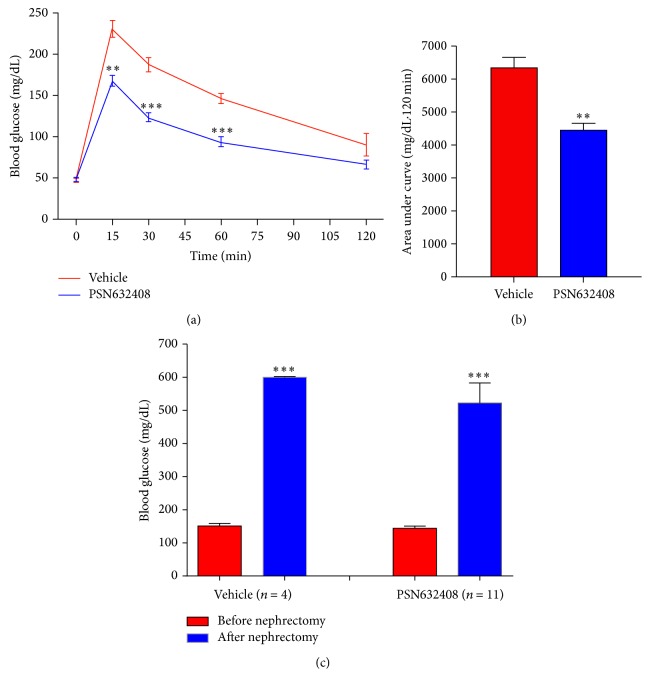
(a) OGTT in PSN632408- or vehicle-treated diabetic mice with normoglycaemia at 4 weeks after transplantation. PSN632408-treated mice (*n* = 9) had significant decreases in glucose levels compared with vehicle-treated mice (*n* = 4) after glucose challenge (^*∗∗*^ *P* < 0.002, ^*∗∗∗*^ *P* < 0.001). (b) Area under curve (AUC_0–120_) during OGTT. AUC_0–120_ was significantly decreased in PSN632408-treated mice (*n* = 9) compared with vehicle-treated mice (*n* = 4) (^*∗∗*^ *P* < 0.002). (c) Blood glucose levels in PSN632408 (*n* = 9) or vehicle-treated diabetic mice (*n* = 4) with normoglycaemia before and after removal of left kidney bearing human islet grafts (^*∗∗∗*^ *P* < 0.001).

To determine if the transplanted mice that achieved normoglycaemia had improved *β*-cell function, an oral glucose tolerance test was performed at 4 weeks after transplantation. PSN632408-treated mice had an increase in blood glucose (168.0 ± 6.7 mg/dL) that was significantly less than in vehicle-treated mice (231.5 ± 10.3 mg/dL) at 15 minutes after glucose challenge (*P* < 0.002). Also, blood glucose levels in PSN632408-treated mice were significantly lower than in vehicle-treated mice at 30 and 60 minutes after glucose load (*P* < 0.001) (Figure 2(a)). The AUC glucose values during the 120 minutes after glucose load were 6,350 ± 320 in PSN632408-treated mice and 4,430 ± 220 in vehicle-treated mice (*P* < 0.002) (Figure 2(b)).

Islet graft function was confirmed by removing the islet bearing left kidneys from mice that achieved normoglycaemia after treatment with PSN632408 or vehicle. After nephrectomy, nonfasting blood glucose was monitored daily without any treatment. All nephrectomized mice either treated with vehicle (*n* = 4) or PSN632408 (*n* = 11) returned back to the diabetic state within 6 days, indicating that achievement of normoglycaemia in the mice could be attributed to function of the human islet graft (Figure 2(c)).

### 3.2. PSN632408 Treatment Increased *β*-Cell Area in Islet Grafts

**Figure 3:**
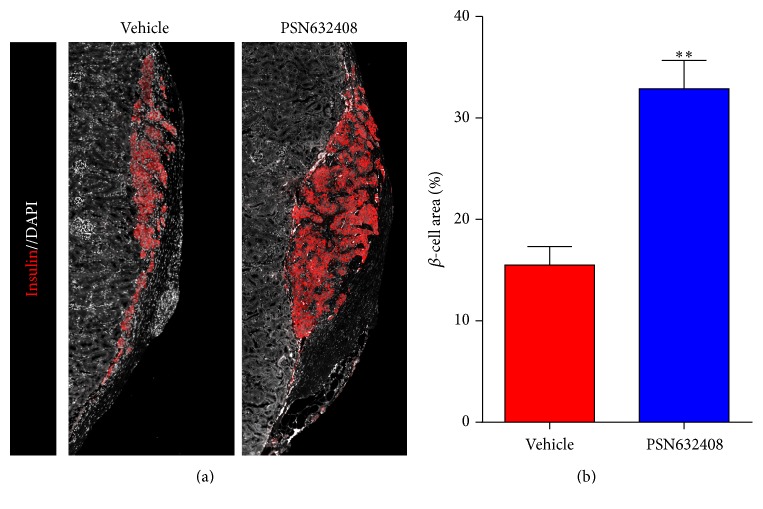
(a) Immunofluorescent staining for insulin (red color) and DAPI (white color) in human islet grafts from the same donor under kidney capsules in PSN632408- and vehicle-treated mice at 4 weeks after transplantation. (b) Percentage of *β*-cell area in islet grafts. *β*-cell area in islet grafts of PSN632408-treated mice (*n* = 6) was significantly increased compared with vehicle-treated mice (*n* = 3) (^*∗∗*^ *P* < 0.001).

*β*-cell area was measured in human islet grafts from PSN632408 or vehicle-treated mice that achieved normoglycaemia after 4 weeks after transplantation. More *β*-cell area was detected in PSN632408-treated mice compared with vehicle-treated mice (Figure 3(a)). The *β*-cell area in islet grafts was 32.8 ± 2.8% in PSN632408-treated mice and 15.4 ± 1.84% in vehicle-treated mice (*P* < 0.001) (Figure 3(b)). These results show that GPR119 agonists could expand the human *β* mass in islet grafts from younger donors.

### 3.3. GPR119 Activation by PSN632408 Stimulates Human *β*-Cell Replication

**Figure 4:**
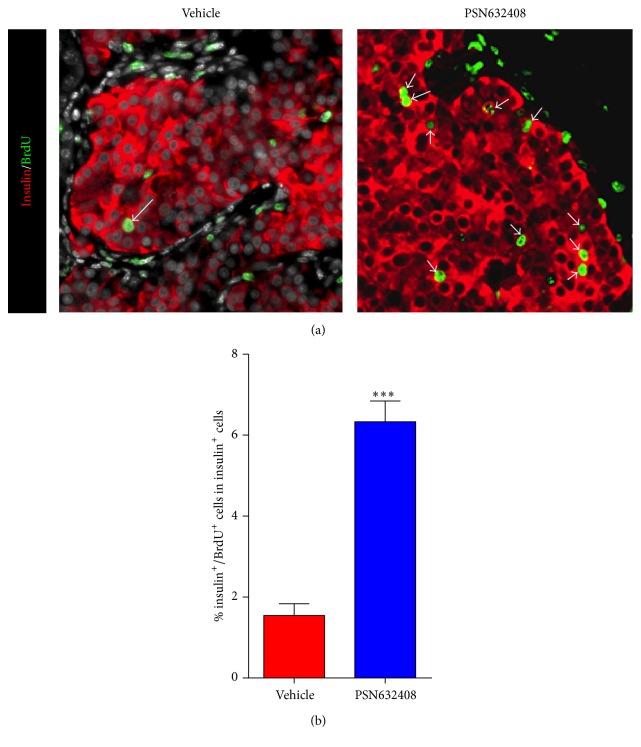
(a) Immunofluorescent staining for insulin (red color) and BrdU (green color) in human islet grafts from PSN632408- and vehicle-treated mice with normoglycemia at 4 weeks after transplantation. White arrows show insulin/BrdU copositive cells in human islet grafts. (b) Percentage of insulin/BrdU copositive cells among total insulin positive cells in human islet grafts. The average percent of insulin/BrdU copositive cells among total insulin positive cells was significantly higher in PSN632408-treated mice (*n* = 9) compared with vehicle-treated mice (*n* = 4) (^*∗∗∗*^ *P* < 0.001).

**Figure 5:**
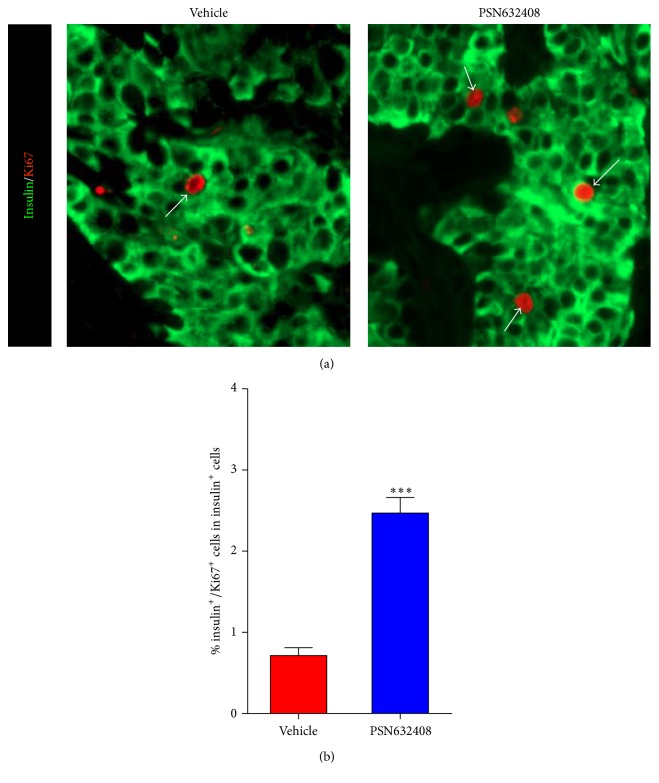
(a) Immunofluorescent staining for insulin (green color) and Ki67 (red color) in human islet grafts from PSN632408- and vehicle-treated mice with normoglycemia at 4 weeks after transplantation. White arrows show insulin/Ki67 copositive cells in human islet grafts. (b) Percentage of insulin/Ki67 copositive cells among total insulin positive cells in human islet grafts. The average percent of insulin/Ki67 copositive cells among total insulin positive cells was significantly higher in PSN632408-treated mice (*n* = 9) compared with vehicle-treated mice (*n* = 4) (^*∗∗∗*^ *P* < 0.001).

To understand the mechanism of expansion of *β*-cell area in human islet grafts, we carried out BrdU labeling to determine the rate of replication of *β*-cells in human islet grafts transplanted in mice treated with PSN632408 or vehicle. Although insulin/BrdU copositive cells were detected in both PSN632408- and vehicle-treated mice, more insulin/BrdU copositive cells were seen in islet grafts in PSN632408-treated mice (Figure 4(a)). The percentage of insulin/BrdU copositive cells was 6.3 ± 0.5% in PSN632408-treated mice and 1.5 ± 0.2% in vehicle-treated mice (*P* < 0.001) (Figure 4(b)). To substantiate this observation, we also performed immunostaining for Ki67, a marker for cellular replication that is incorporated during all phases of the cell cycle. More insulin/Ki67 copositive cells were detected in PSN632408-treated mice (Figure 5(a)). The percentage of insulin/Ki67 copositive cells in human islet grafts was significantly higher in PSN632408-treated mice (2.5 ± 0.2%) compared with vehicle-treated mice (0.7 ± 0.1%, *P* < 0.001) (Figure 5(b)). Taken together, the results of BrdU labeling and Ki67 staining show that activation of GPR119 by PSN632408 can stimulate *β*-cell replication in human islets.

### 3.4. PSN632408 Induces *β*-Cell Neogenesis from Human Duct Cells

**Figure 6:**
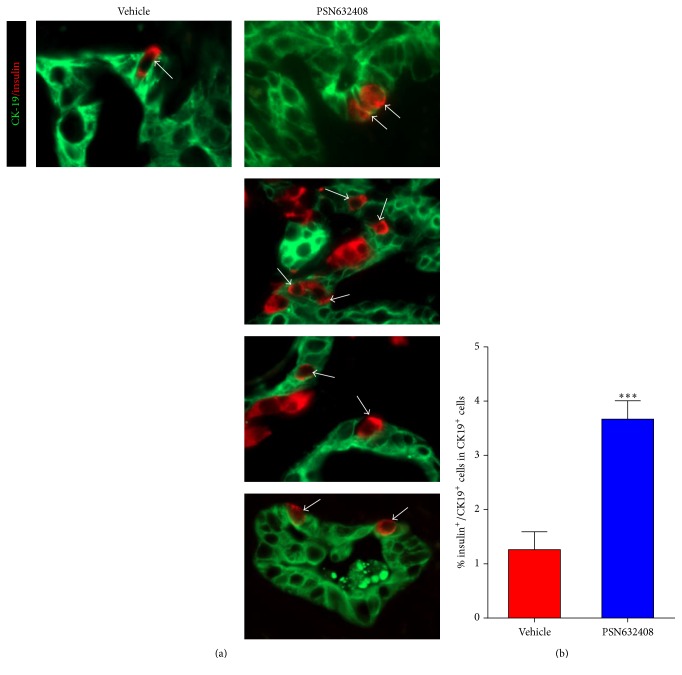
(a) Immunofluorescent staining for insulin (red color) and CK19 (green color) in human islet grafts in PSN632408- and vehicle-treated mice with normoglycaemia at 4 weeks after transplantation. White arrows show insulin/CK19 copositive cells in human islets grafts. (b) Percentage of insulin/CK19 copositive cells among total CK19 positive duct cells in human islet grafts. The average percent of insulin/CK19 copositive cells among total CK19 positive duct cells was significantly higher in mice treated with PSN632408 (*n* = 9) compared with mice treated with vehicle (*n* = 4) (^*∗∗∗*^ *P* < 0.001).

In our previous study, we found that PSN632408 stimulated *β*-cell neogenesis from pancreatic ducts in STZ-diabetic mice [20]. We therefore sought to determine whether PSN632408 can induce *β*-cell neogenesis from isolated human islets that included duct cells. We performed immunostaining for insulin and the pancreatic duct cell marker CK19 in islet grafts. Four weeks after transplantation very few pancreatic acinar cells were left as evidenced by amylase immunostaining (data not shown). In contrast, pancreatic duct cells were abundant and insulin positive cells were detected within the ducts in islet grafts in mice treated with PSN6432408 or vehicle (Figure 6(a)). The percentage of insulin/CK19 copositive cells in ducts in PSN632408-treated mice (3.7 ± 0.3%) was significantly higher than in vehicle-treated mice (1.3 ± 0.3%, *P* < 0.001) (Figure 6(b)). This shows that activation of GPR119 can induce *β*-cell neogenesis from human pancreatic duct cells.

### 3.5. GPR119 Activation by PSN632408 Stimulates Human *α*-Cell Area and Replication

**Figure 7:**
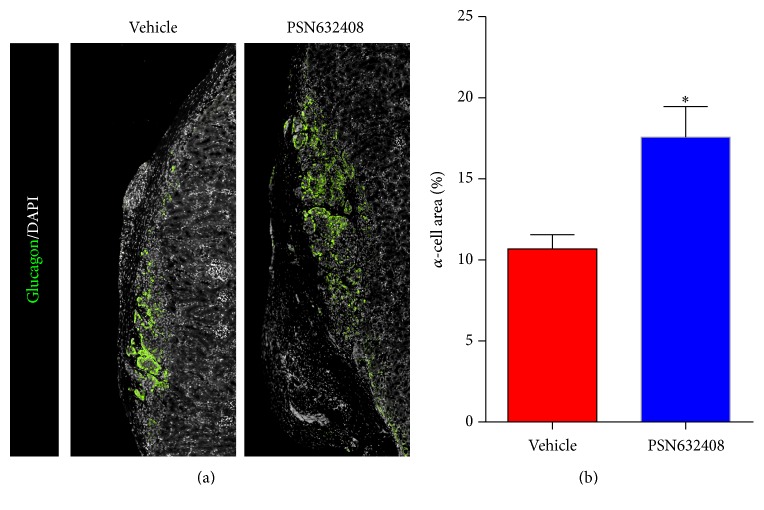
(a) Immunofluorescent staining for glucagon (green color) and DAPI (white color) in human islet grafts from PSN632408- and vehicle-treated mice with normoglycaemia at 4 weeks after transplantation. (b) Percentage of *α*-cell area in human islet grafts. The *α*-cell area in PSN632408-treated mice (*n* = 4) was significantly increased compared with vehicle-treated mice (*n* = 3) (^*∗*^ *P* < 0.02).

**Figure 8:**
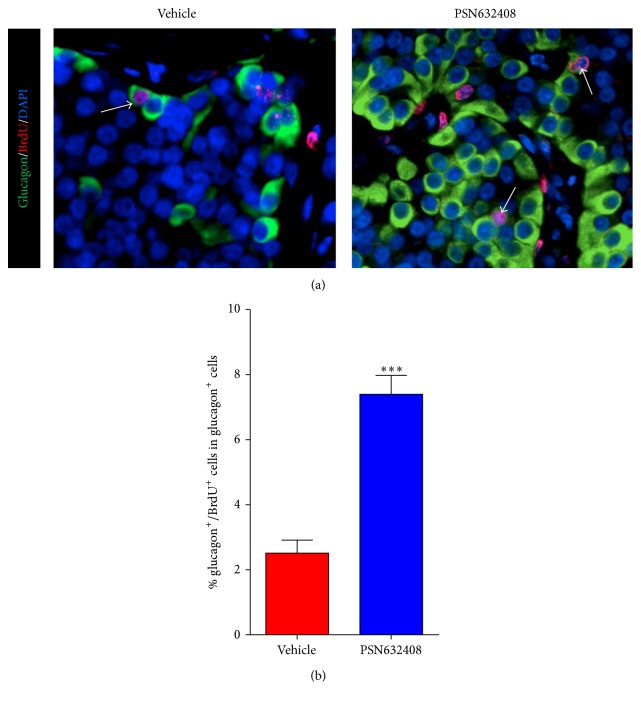
(a) Immunofluorescent staining for glucagon (green color), BrdU (red color), and DAPI (blue color) in human islet grafts from PSN632408- and vehicle-treated mice with normoglycaemia at 4 weeks after transplantation. White arrows show glucagon/BrdU copositive cells in human islet grafts. (b) Percentage of glucagon/BrdU copositive cells among total glucagon positive cells in human islet grafts. The average percent of glucagon/BrdU copositive cells among total glucagon positive cells was significantly higher in PSN632408-treated mice (*n* = 9) compared with vehicle-treated mice (*n* = 4) (^*∗∗∗*^ *P* < 0.001).

We previously reported that PSN632408 treatment increased pancreatic islet *α*-cell mass in STZ diabetic mice [20]. Since human islets are composed of a high percentage of glucagon-containing *α*-cells, we next evaluated the effect of PSN632408 on human *α*-cells. More glucagon positive cells in islet grafts were seen in PSN632408-treated mice (Figure 7(a)). The *α*-cell area in islet grafts was 17.5 ± 1.8% in PSN643408-treated mice and 10.6 ± 0.8% in vehicle-treated mice (*P* < 0.02) (Figure 7(b)). To understand the mechanism of PSN632408 in increasing the *α*-cell mass, we carried out glucagon/BrdU immunostaining of the human islet grafts (Figure 8(a)). Glucagon/BrdU copositive cells were detected more often in PSN632408-treated mice (7.4 ± 0.5%) than in vehicle-treated mice (2.5 ± 0.3%) (*P* < 0.001) (Figure 8(b)).

## 4. Discussion

Since our ability to directly study human *β*-cell function and regeneration in human subjects is limited due to technical and ethical constraints, we have employed humanized mouse models to study human *β*-cell function and regeneration* in vivo* by transplanting human islets into immunodeficient mice [10, 29, 30]. In order to determine the efficacy of PSN632408 on human *β*-cell function and regeneration, we transplanted human islets in diabetic immunodeficient NOD.scid mice. PSN632408 treatment restored normoglycaemia in 71% of mice compared with 24% of mice treated with vehicle at four weeks after transplantation. In addition, oral glucose tolerance in PSN632408-treated mice that returned to normoglycaemia was significantly better than in normoglycemic vehicle-treated mice. This suggests that PSN632408 treatment improved the function and/or mass of *β*-cells in the human islet grafts. In our previous studies with humanized mice, insulin secretagogues (GLP-1, exenatide, glyburide, nateglinide, and sitagliptin) increased human C-peptide secretion and improved oral glucose tolerance [29]. Although we did not determine the levels of human C-peptide in humanized mice in this study, we did remove the human islet grafts to determine graft function. Return of hyperglycemia in recipient mice after islet graft removal indicated that human islet *β*-cell function was essential for maintaining normoglycaemia and improving glucose tolerance.

There are numerous reports on the importance of cell replication as the major source of postnatal *β*-cell expansion [31, 32]. In a previous study, we found that PSN632408 can stimulate pancreatic mouse *β*-cell replication and improve syngeneic islet graft function in mice with STZ-induced diabetes [19]. In another recent study, we demonstrated that PSN632408 treatment increased mouse *β*-cell mass in diabetic mice [20]. In the present study, we observed a significant increase in *β*-cell area in human islet grafts in PSN632408-treated immunodeficient mice with diabetes compared to vehicle-treated immunodeficient mice with diabetes.

To address the mechanism of increase in human *β*-cell area in PSN632408-treated mice, we carried out BrdU immunostaining to measure *β*-cell replication. Mice were continuously labeled with BrdU to determine *β*-cell replication throughout the treatment period. PSN632408 stimulated a significant increase in BrdU incorporation in the human islet graft *β*-cells that were increased fourfold compared with vehicle-treated mice. A recent study reported a low level of human *β*-cell replication in immunodeficient mice transplanted with human islets after treatment with combinations of exendin-4, gastrin, and EGF [28]. One possible reason for low human *β*-cell replication in that study was that the BrdU was administered in the last 24 hours at 8-hour intervals before harvesting grafts from the mice. Adult *β*-cell replication is a slow process; therefore long-term BrdU administration is required to successfully label and track replicating cells during the course of treatment. Since BrdU does not discriminate between replicating *β* cells and cells undergoing DNA repair, BrdU could result in an overestimate of *β*-cell replication [33]. We therefore performed Ki67 immunostaining to validate our observations with BrdU, as Ki67 is expressed during all stages of the cell cycle and is degraded before cells reenter gap (G_0_). Interestingly, we found a significant increase in the percentage of insulin/Ki67 copositive cells in PSN632408 treated mice (2.5 ± 0.2%) compared with vehicle-treated mice (0.7 ± 0.1%). Replicating cells identified by Ki67 immunostaining accounts for cells that replicated within the last 24 hours.

Previous studies have reported that human *β*-replication declines with age [10, 25, 34]. Islets from aged donors result in worse transplantation outcomes and have reduced *β*-cell turnover compared with islets from younger donors [35, 36]. We previously reported that exendin-4 can promote *β*-cell replication in human islets from young donors [10]. Therefore we used pancreases from donors at age of 14 ± 4 years. Since PSN632408 treatment significantly enhanced replication in human *β*-cells from young donors, it would be interesting to assess the same with older donors.

In an investigation carried out using human pancreatic cell preparations with a low purity of islet cells and abundant duct cells, profound *β*-cell neogenesis from duct cells was stimulated with GLP-1 and gastrin [26]. Since PSN632408 can induce *β*-cell neogenesis from duct cells in the diabetic mouse pancreas [20], we hypothesized that PSN623408 might induce *β*-cell neogenesis from human duct cells. We found that PSN632408 stimulated a threefold increase in insulin-stained cells in pancreatic duct cells present in human islet grafts. The signaling pathways that promote duct cell differentiation and *β*-cell neogenesis are not clearly understood. Activation of the transcription factor Neurogenin 3 in cells within or adjacent to the pancreatic ducts promotes *β*-cell neogenesis in mice [37]. Studies are needed to further determine the mechanism of human *β*-cell neogenesis.

In addition to *β*-cell replication and neogenesis, prevention of *β*-cell apoptosis may have also contributed to the increase of PSN632408-induced *β*-cell area in the human islet grafts. At 4 weeks after transplantation, very few apoptotic *β*-cells were detected by TUNEL immunostaining (data not shown). Therefore, it cannot be ruled out that PSN632408 treatment could have prevented early apoptosis after transplantation and contributed to the increase in total *β*-cell area. A time course study of apoptosis after transplantation would be required for definitive evidence to evaluate the antiapoptotic effects of PSN632408.

In addition to an increase in human *β*-cell area, we found a significant increase in human *α*-cell area in PSN632408-treated mice compared with vehicle-treated mice. Increases in *α*-cells and elevated glucagon secretion may aid in the formation of new *β*-cells, since pancreatic glucagon can induce *β*-cell differentiation [38]. It was recently reported that *α*-cells can regenerate and convert into insulin-producing *β*-cells through the ectopic expression of a single gene, Pax4 [39]. However, these studies were limited to murine models. We do not have evidence showing conversion of human *α*-cells to human *β*-cells and whether it would occur in humans needs to be further explored.

PSN632408 can increase intracellular cAMP levels in a GPR-119 dependent manner. We recently found that PSN632408 can stimulate *β*-cell replication in mouse islets* in vitro* and* in vivo* and can increase plasma active GLP-1 levels in mice. Therefore, it will be interesting to further compare the effect of GPR119 agonists with GLP-1 and its analogues and determine whether PSN632408 may improve human islet function and stimulate human *β*-cell regeneration either through direct activation of human *β* cells or indirectly by stimulating GLP-1 secretion.

The islet mass from single donors is often not sufficient to restore normoglycemia in islet transplanted patients. Our studies provide evidence that GPR119 activation can stimulate human *β*-cell regeneration. Therefore, GPR119 agonists may qualify as therapeutic agents to regenerate *β*-cells and to increase *β*-cell mass in islet transplanted patients with type 1 diabetes.
